# Outcome of endoscopy-negative iron deficiency anemia in patients above 65: A longitudinal multicenter cohort

**DOI:** 10.1097/MD.0000000000005339

**Published:** 2016-11-28

**Authors:** Raphaël Clere-Jehl, Erik Sauleau, Stefan Ciuca, Mickael Schaeffer, Amanda Lopes, Bernard Goichot, Thomas Vogel, Georges Kaltenbach, Eric Bouvard, Jean-Louis Pasquali, Daniel Sereni, Emmanuel Andres, Anne Bourgarit

**Affiliations:** a Internal Medicine, Endocrinology and Nutrition Department, Hautepierre Hospital; b Medical Information and Statistics Department, Civil Hospital, University Hospital of Strasbourg, Strasbourg; c Internal Medicine Department, Saint-Louis Hospital; d Internal Medicine Department, Lariboisière Hospital, APHP, University Hospital of Paris, Paris; e Geriatric Department, Robertsau Hospital, University Hospital of Strasbourg, Strasbourg; f Acute Gerontology Department, Tenon Hospital, APHP, University Hospital of Paris, Paris; g Internal Medicine Department, New Civil Hospital; h Internal Medicine Department, Civil Hospital, University Hospital of Strasbourg, Strasbourg, France.

**Keywords:** diagnosis, elderly, endoscopy-negative, iron deficiency anemia, multicenter, outcome

## Abstract

After the age of 65 years, iron deficiency anemia (IDA) requires the elimination of digestive neoplasia and is explored with upper and lower gastrointestinal (GI) endoscopy. However, such explorations are negative in 14% to 37% of patients. To further evaluate this issue, we evaluated the outcomes of patients aged over 65 years with endoscopy-negative IDA.

We retrospectively analyzed the outcomes of in-patients over the age of 65 years with IDA (hemoglobin <12 g/dL and ferritin <70 μg/L) who had negative complete upper and lower GI endoscopies in 7 tertiary medical hospitals. Death, the persistence of anemia, further investigations, and the final diagnosis for IDA were analyzed after at least 12 months by calling the patients’ general practitioners and using hospital records.

Between 2004 and 2011, 69 patients (74% women) with a median age of 78 (interquartile range (IQR) 75–82) years and hemoglobin and ferritin levels of 8.4 (IQR 6.8–9.9) g/dL and 14 (IQR 8–27) μg/L, respectively, had endoscopy-negative IDA, and 73% of these patients received daily antithrombotics. After a follow-up of 41 ± 22 months, 23 (33%) of the patients were dead; 5 deaths were linked with the IDA, and 45 (65%) patients had persistent anemia, which was significantly associated with death (*P* = 0.007). Further investigations were performed in 45 patients; 64% of the second-look GI endoscopies led to significant changes in treatment compared with 25% for the capsule endoscopies. Conventional diagnoses of IDA were ultimately established for 19 (27%) patients and included 3 cancer patients. Among the 50 other patients, 40 (58%) had antithrombotics.

In endoscopy-negative IDA over the age of 65 years, further investigations should be reserved for patients with persistent anemia, and second-look GI endoscopy should be favored. If the results of these investigations are negative, the role of antithrombotics should be considered.

## 1 Introduction

Iron deficiency anemia (IDA) is the leading cause of anemia worldwide^[1,2]^ and is one of the top causes of anemia among patients aged over 65 years in developed countries.^[3–5]^ Among these patients, 63% to 86% of the cases of IDA are due to bleeding bowel lesions,^[6–12]^ which has led to recommendations for systematic upper and lower gastrointestinal (GI) endoscopy procedures for IDA exploration.^[13–15]^ However, 14% to 37% of these endoscopic procedures are not able to determine the cause of the iron deficiency.^[7,8,16]^ In these cases, which are also called “obscure bleeding”,^[17]^ the guidelines recommend further investigations for men and postmenopausal women with persistent anemia.^[14,15]^

In patients aged older than 65 years, the efficiency of these further explorations may be better due to higher frequencies of cancers^[18]^ and angiodysplasia diagnoses.^[19]^ However, this improved efficacy should be balanced by the worsening of the side effects of these explorations in elderly patients.^[20]^ Moreover, no specific guidelines are available for patients aged over 65 years.

To evaluate the accuracy of the initial endoscopies, and thus the potential need for further explorations, we retrospectively evaluated the clinical outcomes and final diagnoses of IDA in patients aged over 65 years for 1 year or more after an initial negative standard digestive examination for IDA.

## 2 Methods

### 2.1 Patients

All in-patients aged over 65 years who had undergone upper and lower GI endoscopy between 2004 and 2011 in 7 internal medicine units of 3 French university hospitals were retrospectively considered.

To be included in the outcome study, the patients had to meet the following eligibility requirements: proven IDA as defined by a hemoglobin level below 12 g/dL and a serum ferritin level below 70 μg/L; no significant initial GI lesion known to lead to IDA, no cancer of any part of the explored bowels (i.e., the esophagus, stomach, colon, and rectum), no esophagitis, no ulcers of the esophagus, stomach, or duodenal ulcers, no angiodysplasia of any explored part, no stomach and colon polyps greater than 15 mm, and no inflammatory bowel disease; and the patients must have had a minimum of 12 months of follow-up.

Patients with a known active chronic pathology potentially inducing severe anemia were excluded: end-stage kidney disease (glomerular filtration rate inferior to 15 mL/min), hemoglobinopathies such as thalassemia, hematological malignancy, aplastic anemia, metastatic cancer or autoimmune diseases resulting in anemia: autoimmune hemolytic anemia, Biermer disease, and systemic lupus erythematosus.

Our study received the approval of the ethics committee of the University Hospital of Strasbourg in April 2015.

### 2.2 Baseline characteristics

The patients’ epidemiologic characteristics and treatments that potentially induced bleeding, including nonsteroidal anti-inflammatory drugs (NSAIDs), antiplatelets, and anticoagulants were retrospectively recorded. NSAIDs and aspirin (even at doses from 75 to 300 mg/d) were considered to be gastrotoxic drugs. Hemoglobin, mean corpuscular volume (MCV), serum ferritin, and transferrin saturation (TSAT) were recorded.

Minimal digestive lesions observed during the initial endoscopic procedures that were not considered to be the sole causes of the IDA were registered (e.g., hiatal hernia, colonic diverticulosis, nonerosive gastritis, hemorrhoidal diseases, and colonic polyps smaller than 15 mm).

### 2.3 Outcome

Information about the outcomes was collected between 2010 and 2014 via telephone calls to the general practitioners of the patients and/or by analyzing the medical records in cases of new in-patient stays. The following data about the patients’ outcomes were registered: survival, cause of death (and link with anemia), persistent anemia and/or long-term iron supplementation, and transfusions. The investigations performed after the first standard endoscopic procedure and their delays were registered (termed “early further investigations” when performed in the first 3 months following inclusion and “follow-up investigations” when delayed) as were the diagnoses of IDA and modifications of therapy. The final diagnosis regarding the cause of IDA after at least 1 year of follow-up or at the time of death was noted for each patient.

### 2.4 Statistics analysis

Quantitative variables are described using position and dispersion statistics as the means ± the standard deviations, medians (IQR 25–75), and confidence intervals. Qualitative variables are described as numbers and percentages. Comparisons between the quantitative variables were realized with Student *t* tests, ANOVAs or nonparametric Mann–Whitney Wilcoxon/Kruskal–Wallis tests. To compare the qualitative variables, chi-squared tests or nonparametric Fisher exact tests were used. Survival analyses were realized using Kaplan–Meier curves, and comparisons between groups were performed with log-rank tests. Multivariate logistic regressions were also performed to examine the predictive factors for mortality and anemia. A backward stepwise variable selection procedure was used to avoid redundant information and to select the influential predictive variables in each model. The significance level was fixed at 5%.

The analyses were performed with the R software version 3.1 (Language for Environment and Statistical computing, R Core team, Vienna, Austria) with all requiring additional packages.

## 3 Results

### 3.1 Inclusion

**Figure 1:**
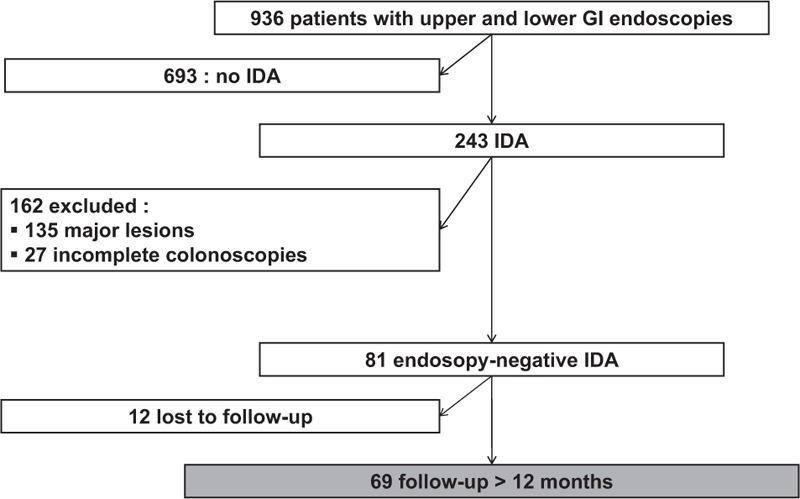
Flowchart. GI = gastrointestinal, IDA = iron deficiency anemia.

Between January 2004 and December 2011, among the 936 in-patients who underwent a GI endoscopic procedure (Fig. 1) in 7 tertiary internal medicine departments, 81 had endoscopy-negative IDA. Among these patients, follow-ups of at least 12 months were ultimately obtained for 69 (85%).

### 3.2 Baseline

The patients’ median age was 78 years (IQR 65–88), 74% (n = 51) were women and they had median baseline hemoglobin, MCV, and ferritin levels of 8.4 g/dL (IQR 6.8–9.9), 79 μm^3^ (IQR 69–85), and 14 μg/L (IQR 8–27), respectively (Table 1). Seventy-five percent (n = 52) were subjected to least 1 treatment that could potentially induce bleeding, including vitamin K antagonists (n = 21), platelet inhibitors (n = 24), both (n = 2), and NSAIDs (n = 5).

**Table 1:**
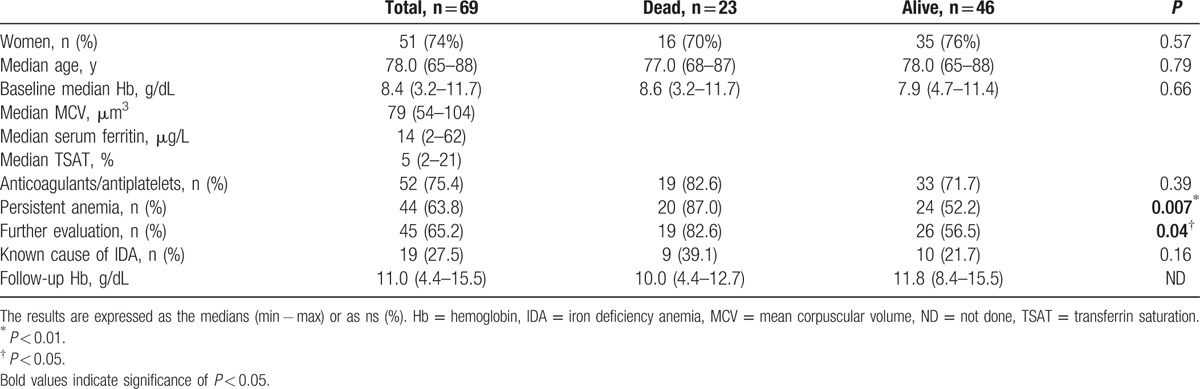
Patients’ characteristics at baseline and survival comparison.

### 3.3 Persistent anemia

After a median follow-up of 40 months (IQR 25–58), the median hemoglobin reached 11.0 g/dL (IQR 10.0–12.3), and 64% (n = 44) of the patients had persistent anemia (hemoglobin [Hb] < 12 g/dL).

Thirty-two percent (n = 22) received at least 1 red cell transfusion, and 51% (n = 35) were subjected to long-term oral iron supplementation. No patient underwent parenteral iron supplementation.

The persistence of anemia was not associated with age, sex, initial ferritin level, severity of the initial anemia, or the administration of “bleeding treatments” but was significantly associated with the following factors: the implementation of further evaluation (77% [n = 34] in cases of persistent anemia vs 44% [n = 11] in cases of resolved anemia; odds ratio (OR) 4.22 confidence interval (IC)95 [1.33–14.3]; *P* = *0.008*); the final discovery of a conventional cause of IDA (36% [n = 16] vs 12% [n = 3]; OR 4.11 IC95 [0.99–24.8]; *P* = *0.05*); and death (46% [n = 20] vs 12% [n = 3]; OR 0.17 IC95 [0.03–0.68]; *P* = *0.007*) (Table 2).

**Table 2:**
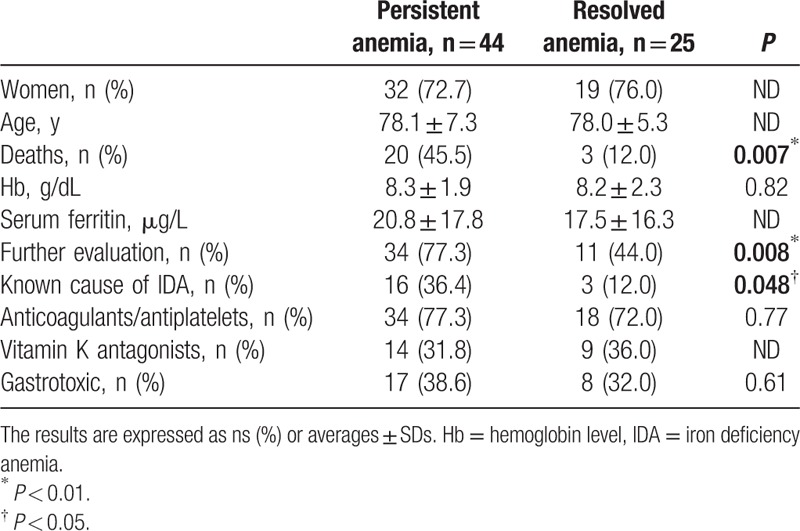
Comparison of patients with and without persistent anemia.

All the 3 patients diagnosed with malignant lesions during the follow-up had persistent anemia.

### 3.4 Death

At the end of the follow-up, 33% (n = 23) of the 69 included patients were dead within a median of 27 months (IQR 16–49). Only 22% (n = 5) of these 23 deaths were considered to be directly linked with IDA. Three of these deaths were caused by malignant lesions, including colon carcinomas (n = 2) and bladder cancer with macroscopic hematuria aggravated by anticoagulants (n = 1), and 2 were due to digestive hemorrhages resulting from nonmalignant lesions. The 18 remaining deaths were mostly due to the following causes: cardiovascular causes (n = 6), sepsis (n = 4), nonbleeding cancers including lung cancer (n = 1) and myeloma (n = 1). Apart from the persistence of anemia, the other factor that was significantly associated with death was further investigations, which were performed in 83% of the patients who died versus 65% of the survivors (OR 3.6 IC95 [0.98–16.8], *P* = *0.04*; Table 1).

The multivariate analysis revealed that only the persistence of anemia was independently associated with death.

### 3.5 Further explorations

**Figure 2:**
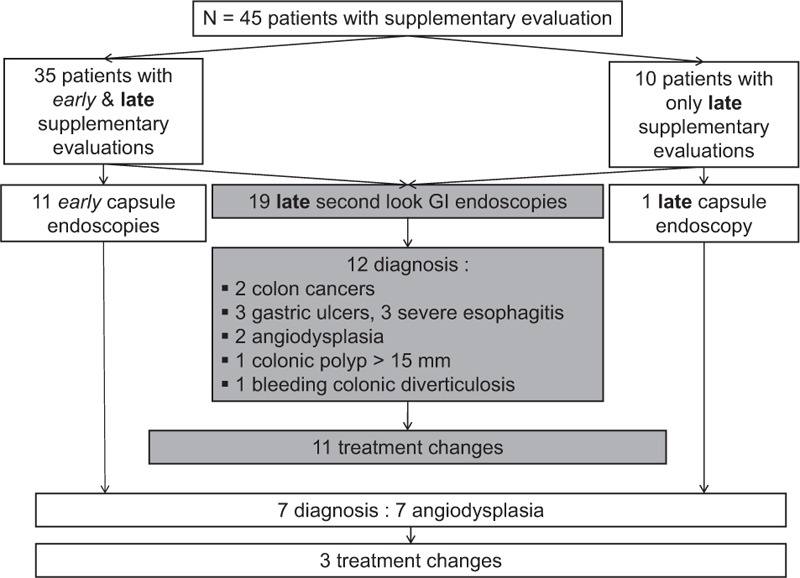
Time of supplementary evaluation with focus on capsule endoscopies and second-look endoscopies’ efficiency. GI = gastrointestinal.

Among the 69 patients with follow-up data, 65% (n = 45) underwent at least 1 further investigation (Fig. 2); 51% (n = 35) of these explorations were “early” further explorations (<6 months after IDA diagnosis) and 14% (n = 10) were performed later as “follow-up” investigations. The only factor that was significantly associated with the specifics of the further investigations was the extent of the baseline anemia (Hb 7.8 g/dL [IQR 6.7–9.6] for the early-explored patients vs 9.6 g/dL [IQR 9.5–10.4] for the late-explored patients; *P* = *0.01*).

Among these 45 patients, 77 further explorations were performed and included the following: second-look GI endoscopies (n = 19), capsule endoscopies (n = 12), abdominal CTs (n = 14), abdominal US scans (n = 17), gynecologic exams (n = 7), and small bowel barium enemas (n = 6).

Among the 19 second-look upper and/or lower GI endoscopies, all were performed at follow-up, 12 (63%) led to causal diagnoses for IDA, and 11 (58%) led to significant treatment changes. The 2 colon cancer diagnoses were preformed via second-look lower endoscopy after 6 and 11 months of follow-up.

Twelve capsule endoscopies were performed, and these procedures were most frequently performed within the first 3 months following inclusion (early further explorations) and led to causal diagnoses for IDA in 7 cases (58%), all of which were angiodysplasias. These diagnoses resulted in treatment changes for 3 patients (2 enteroscopy procedures and 1 vitamin K antagonist removal).

Eight patients were tested for coeliac disease (antibodies and biopsies), and all were negative.

Although the mortality rate was significantly higher in the patients who underwent further evaluations during follow-up, there was no direct complication of the further investigations that led to death.

### 3.6 IDA causes

**Figure 3:**
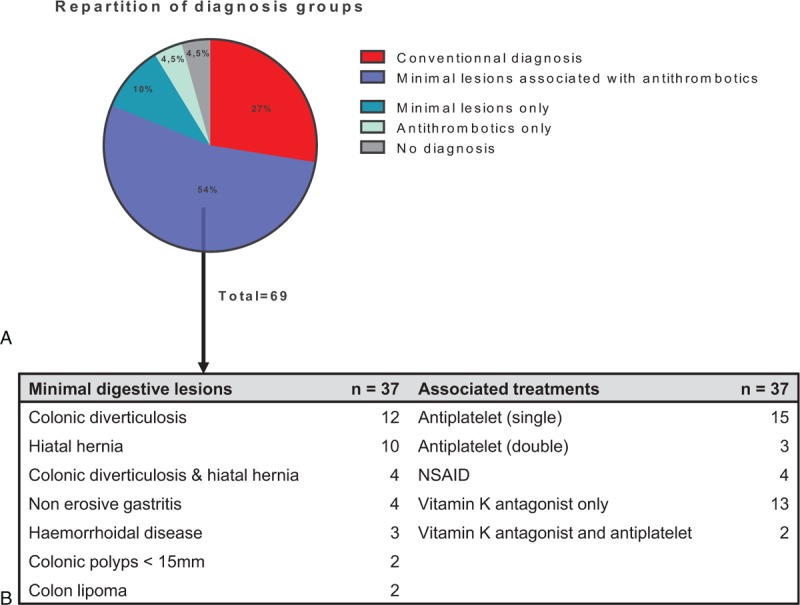
(A) Rediagnoses after follow-up in 69 patients. (B) Types of digestive lesions and types of associated antithrombotic drugs. NSAID = nonsteroidal anti-inflammatory drug. The black arrow points out the details in the largest group: minimal lesions associated with antithrombotics.

At the end of the study follow-up, conventional causal diagnoses were established for 27% (n = 19) of patients (Fig. 3) and included malignancy in 3 patients.

The remaining 73% (n = 50) of patients achieved no causal IDA diagnoses by the end of follow-up. Among the 50 patients with no conventional causal IDA diagnoses, 80% (n = 40) received daily antithrombotics, 74% (n = 37) had an association of antithrombotics with minimal lesions. In this group, the most common minimal lesions were colonic diverticulosis (n = 12), hiatal hernia (n = 10), associated colonic diverticulosis and hiatal hernia (n = 4), and nonerosive gastritis (n = 4). Most of the antithrombotic drugs were platelet inhibitors. Among the remaining 13 patients without causal IDA diagnoses by the end of follow-up, 7 patients only had a minimal digestive lesion and were not on antithrombotics, whereas 3 patients only had antithrombotics without any lesion and 3 patients had no antithrombotic and no lesion at all.

### 3.7 Subgroups

To evaluate the roles of very old age and severe anemia, we compared subgroups and did not find any significant differences in death, further investigation rate, final diagnosis in the patients older than 80 years, or those with hemoglobin levels below 9 g/dL.

## 4 Discussion

Our study revealed that in patients with endoscopy-negative IDA above the age of 65 years, the large majority of whom were treated with antithrombotic drugs, conventional causal diagnoses were finally established for 27%. In these patients, anemia persisted in 64%, and this persistence was significantly associated with death, further investigations, and the ultimate final discovery of a conventional cause of IDA.

To our knowledge, this study is the first to exclusively include patients aged over 65 years.^[21–24]^ Furthermore, only in-patients were included because we wanted to focus on patients with higher comorbidities. However, the eligibility of the patients for upper and lower GI endoscopies represents a selection bias. Overall, this bias can probably explain the high rate of patients on antithrombotic drugs compared with others (73% vs 48%^[22]^), the high frequency of persistent anemia (64% vs 42%^[22]^ to 51%),^[21]^ and the mortality (33% vs 20% to 25%).^[21,22,24]^ However, the 33% rate of endoscopy-negative IDA was not different from the results of previous studies, which have reported rates from 14% to 37%.^[7,8,16]^

As in other studies, the deaths were not predominantly linked with the IDA cause but rather with cardiovascular diseases. Notably, the deaths were not linked to the investigations.

We confirmed that persistent or recurrence of anemia was significantly associated with a worse prognosis,^[21]^ particularly death (OR 0.17 [0.03–0.68]), but it was also associated with the final discovery of a cause of the IDA. As noted in the study by Soon et al,^[21]^ this finding might be attributable to the fact that the further investigations were mostly performed or the patients with persistent or recurrent anemia according to the American guidelines.

Because the French guidelines do not limit further investigations to patients with persistent anemia, 44% of the patients with resolved anemia also underwent further investigations in our study, which allowed us to retrospectively evaluate their usefulness.

Moreover, we underline the relatively good prognoses of the nonexplored patients, who exhibited significantly lower mortality, which indicates that abstention may also be a safe decision.

As in other studies, we found that only capsule endoscopies and second-look endoscopies enabled IDA diagnoses and that the endoscopies elicited greater effects (treatment changes). Notably, in our study, capsule endoscopies allowed only for the diagnoses of benign lesions, all of which were angiodysplasias, that are more frequent in the older patients.^[19]^ The efficacies of specific treatments for angiodysplasias remain controversial.^[25–28]^

Regarding the final diagnoses of IDA, 27% of patients had a final conventional cause of IDA that had been missed after in first standard exploration. The only factor associated with this 27% rate of diagnosis was persistent anemia. This finding allows us to support the proposal of others that persistent anemia should lead to further explorations even in old patients. Interestingly, we did not observe any differences in outcome in the oldest or most severely anemic patients.

After no conventional cause could be found, we observed that the majority of these patients received daily antithrombotics. Most patients had an association between a minimal digestive lesion and the use of antithrombotic drugs. No previous study has described the association between minimal digestive lesions and antithrombotics or proposed this association as a possible cause of IDA. This association could be explained by the high frequencies of both among older patients.^[21]^ Bleeding medication certainly may be the cause of obscure bleeding but this is more likely to come from a noninvestigated area or missed lesion (as the paper has shown), and not be due to the association with finding a minimal diagnostic lesion. Because this study was retrospective, this mechanism is uncertain and its identification would require further prospective studies.

Altogether, our results allow us to suggest that in cases of endoscopy-negative IDA in patients over the age of 65 years, further explorations should be reserved for patients with recurrent or persistent anemia according to the guidelines^[14]^ and that in these cases, second-look endoscopy appears to be the exploration of choice. Moreover, antithrombotic drugs may induce or increase an obscure bleeding in this population.

## 5 Conclusion

In endoscopy-negative IDA in patients over the age of 65 years, further investigations should be reserved for patients with persistent anemia, and second-look GI endoscopy should be favored in this situation. If a second-look GI endoscopy is negative, the role of antithrombotics should be considered.
